# Classifying Charge Carrier Interaction in Highly Compressed Elements and Silane

**DOI:** 10.3390/ma14154322

**Published:** 2021-08-02

**Authors:** Evgueni F. Talantsev

**Affiliations:** 1 Department of Precision Metallurgy and Pressure Processing Technologies, M. N. Mikheev Institute of Metal Physics, Ural Branch, Russian Academy of Sciences, 18, S. Kovalevskoy Street, 620108 Ekaterinburg, Russia; evgeny.talantsev@imp.uran.ru; Tel.: +7-912-676-0374; 2 NANOTECH Centre, Ural Federal University, 19 Mira Street, 620002 Ekaterinburg, Russia

**Keywords:** superconductivity induced by high-pressure, charge carrier interaction in superconductors, non-electron–phonon mediated superconductivity

## Abstract

Since the pivotal experimental discovery of near-room-temperature superconductivity (NRTS) in highly compressed sulphur hydride by Drozdov et al. (*Nature* **2015**, *525*, 73–76), more than a dozen binary and ternary hydrogen-rich phases exhibiting superconducting transitions above 100 K have been discovered to date. There is a widely accepted theoretical point of view that the primary mechanism governing the emergence of superconductivity in hydrogen-rich phases is the electron–phonon pairing. However, the recent analysis of experimental temperature-dependent resistance, *R*(*T*), in H_3_S, LaH_x_, PrH_9_ and BaH_12_ (Talantsev, *Supercond. Sci. Technol.* **2021**, *34*, accepted) showed that these compounds exhibit the dominance of non-electron–phonon charge carrier interactions and, thus, it is unlikely that the electron–phonon pairing is the primary mechanism for the emergence of superconductivity in these materials. Here, we use the same approach to reveal the charge carrier interaction in highly compressed lithium, black phosphorous, sulfur, and silane. We found that all these superconductors exhibit the dominance of non-electron–phonon charge carrier interaction. This explains the failure to demonstrate the high-*T*_c_ values that are predicted for these materials by first-principles calculations which utilize the electron–phonon pairing as the mechanism for the emergence of their superconductivity. Our result implies that alternative pairing mechanisms (primarily the electron–electron retraction) should be tested within the first-principles calculations approach as possible mechanisms for the emergence of superconductivity in highly compressed lithium, black phosphorous, sulfur, and silane.

## 1. Introduction

Th_4_H_15_ was the first superhydride. Its discovery by Satterthwaite and Toepke [1] was based upon their pivotal idea [1]: “…There has been theoretical speculation [2] that metallic hydrogen might be a high-temperature superconductor, in part because of the very high Debye frequency of the proton lattice. With high concentrations of hydrogen in the metal hydrides one would expect lattice modes of high frequency and if there exists an attractive pairing interaction one might expect to find high-temperature superconductivity in these systems also.”

Nearly twenty superconducting superhydride phases have been discovered [3,4,5,6,7,8,9,10] since the milestone report by Drozdov et al. [3] on the observation of the superconducting transition above 200 K in highly compressed sulfur hydride H_3_S [3]. Despite the wide consensus, supported by first-principles calculations, that the primary mechanism governing near-room-temperature superconductivity (NRTS) in superhydrides is the electron–phonon pairing [9,10] (which is exact idea proposed by Satterthwaite and Toepke in 1970 [1]), there are several dramatic failures of this approach. 

For instance, we can highlight the prediction by Feng et al. [11] who calculated the Debye temperature of $T_{\theta }=3500–4000\;K$ and $T_{c}≅165\;K$ for highly compressed hydrogen-rich silane, SiH_4_, for which the experiment performed by Eremets et al. [12] showed the onset of superconducting transition $T_{c}^{onset}=7–17\;K$ at a pressure varying within in the wide range of 60 GPa≤P≤192 GPa. Talantsev [13] deduced the Debye temperature of $T_{\theta }=353\pm 3\;K$ by fitting experimental *R*(*T*) data for SiH_4_ (compressed at *P* = 192 GPa and exhibited $T_{c}^{onset}≅11\;K$) to the Bloch–Grüneisen (BG) equation [14,15]:

(1) $$R\left(T\right)=R_{0}+A\cdot {\left(\frac{T}{T_{\theta }}\right)}^{5}\cdot \overset{\frac{T_{\theta }}{T}}{\underset{0}{\int }}\frac{x^{5}}{\left(e^{x}-1\right)\cdot \left(1-e^{-x}\right)}\cdot dx$$

where *R*_0_ is the residual resistance at T→0 K due to the scattering of electrons on the static defects of the crystalline lattice (this type of charge carrier was confirmed in direct experiment for NRTS H_3_S [16]), and the second term describes the electron–phonon scattering, where *A* and $T_{\theta }$ are free-fitting parameters. Thus, experimentally observed $T_{c}^{onset}=7–17\;K$ and deduced $T_{\theta }=353\pm 3\;K$ agree well with the weak-coupling scenario, $\frac{T_{c}^{onset}}{T_{\theta }}=0.03$, but both of these values are different from computed ones [11] by more than one order of magnitude. 

It should be stressed that the failure of first-principles calculations for one of the chemically simplest hydrogen-rich superconductors has been at the same unexplained and uncommented status since 2008 [9,10,11,12,17]. 

There are several nearly identical failures of first-principles calculations for low-Z elements; we can mention, for example, highly compressed lithium, with a predicted $T_{c}=50–90\;K$ [18] and exact prediction $T_{c}=55\;K$ at P=40 GPa [18]. Experiments show a small drop (about 5% from normal state resistance) at $T_{c}^{onset}\sim 7\;K$ at a pressure of 22 GPa ≤ *P* ≤ 32 GPa [19]. Shimizu et al. [20] also reported a small drop in resistance $T_{c}^{onset}\sim 6\;K$ at P=40 GPa, while Struzhkin et al. [21] reported the diamagnetic signal at $T_{c}^{onset}\sim 10\;K$. It should be highlighted that, for one sample compressed at *P* = 48 GPa, Shimizu et al. [20,22] reported $T_{c}^{onset}≅20\;K$, while other samples exhibited $6\;K\leq T_{c}^{onset}\leq 10\;K$ at a wide pressure range of 23 GPa ≤P≤80 GPa. Deemyad et al. [23], Matsuoka et al. [24] and Schaeffer et al. [25] reported $T_{c}^{onset}\leq 14\;K$ for lithium compressed at a pressure range of 16 GPa ≤P≤60 GPa. Even the most advanced first-principles calculations [26] did not reproduce *a priori* a known experimental $T_{c}^{onset}\left(P>30\;\mathrm{GPa}\right)$ dataset for highly compressed lithium. 

Extended reviews of the pressure effect on the transition temperature of elements were produced by Shimizu et al. [22] and Buzea and Robbie [27]. In these reviews, basic properties of highly compressed elemental superconductors can be found. It should be noted that elemental sulfur was the first dielectric element which was converted into a superconductor by high pressure, as reported by Yakovlev et al. [28]. The first prediction of the superconducting transition temperature in highly compressed sulfur by first-principles calculations was reported by by Zakharov and Cohen [29], who calculated *T*_c_ = 15 K at *P* > 550 GPa. The superconducting state at lower pressures has not been predicted. Experiments [30,31] showed *T*_c_ = 10–17 K in this element, but at a much lower pressure range of 93 GPa < *P* < 157 GPa. Later, Rudin and Liu [32] were able to show that first-principles calculations can reproduce experimentally observed *T*_c_ in given pressure range, and more recently Whaley-Baldwin et al. [33] reported the results of first-principles calculations for elemental sulfur in a wide pressure range of 250 GPa < *P* < 700 GPa. The highest *T*_c_ = 26.5 K was calculated at *P* = 271 GPa [33], which is far above reported to-date experimentally reachable pressure for this element [22,28,30,31]. Since the superconducting state of this element remains an interest for researchers working in the first-principles calculations field, we here perform an analysis of experimental *R*(*T*) curves for highly compressed sulfur, with the purpose of revealing the charge carrier interaction in this historically first dielectric element, which was converted into a superconductor by high pressure [28]. 

Phosphorus is another element which can be converted into a superconductor by applying high pressure [34]. Wittig and Matthias reported *T*_c_ = 4.7 K [34] for this element when subjected to *P* ~ 10 GPa. Since the superconducting state of this element in a high-pressure condition is still under intensive theoretical and experimental investigation [35,36,37], we here report results of the revealed charge carrier interaction in this highly compressed superconducting element through the analysis of *R*(*T*) data. 

It should be stressed that the electron–phonon pairing [38,39], which is the only pairing mechanism for the superconducting state, and is considered at the moment by first-principles calculations, is not the only mechanism which can cause the formation and the condensation of Cooper pairs. For instance, Matthias [40] and, more recently, Harshman and Fiory [41], and very recently, Kim [42], proposed theories of superconductivity (and for NRTS materials in particular) based on the electron–electron retractive pairing (extended review of other pairing mechanisms is given elsewhere [43]). Non-electron–phonon theories of superconductivity can be partially supported by the recent report [44], where the analysis of the temperature-dependence of the resistivity, *R*(*T*), in superconducting highly compressed superhydrides (H_3_S, LaH_x_, PrH_9_ and BaH_12_) showed that all these materials exhibit the dominance of non-electron–phonon charge carrier interaction in their normal state.

Due to the fact that there is an apparent debate about the primary mechanism for the emergence of NRTS in highly compressed hydrides [9,10,41,42,44], we here aimed to extend this discussion to a wider class of highly compressed superconductors. Namely, to include elemental superconductors and the first polyhydride, SiH_4_, where the superconducting state was induced by high pressure, but where experimentally observed *T*_c_ was more than one order of magnitude lower than the value predicted as based on the electron–phonon phenomenology [11,12]. Thus, we here analyzed temperature-dependent resistance data, *R*(*T*), by the same approach as in Reference [44] to reveal the dominant charge carrier interaction in highly compressed lithium, black phosphorous, sulfur, and silane. In our results, we found that all these superconductors exhibit the dominance of non-electron–phonon charge carrier interaction and that, in particular, these materials exhibit the dominance of electron–electron interaction. Thus, at least partially, the failure of first-principles calculations to predict the superconducting transition temperature in these materials, based on a presumption of electron–phonon mediated superconductivity, can be explained by a different physical mechanism, rather than on more complicated first-principles calculations which were based on already-known experimental results. 

## 2. Model Description

Jiang et al. [45] and later Talantsev [44,46] proposed to reveal the type of the charge carrier interaction in metallic substances using a generalized version of the Bloch–Grüneisen equation [44,45,46]:

(2) $$R\left(T\right)=R_{0}+A_{p}\cdot {\left(\frac{T}{T_{\omega }}\right)}^{p}\cdot \overset{\frac{T_{\omega }}{T}}{\underset{0}{\int }}\frac{x^{p}}{\left(e^{x}-1\right)\cdot \left(1-e^{-x}\right)}\cdot dx$$

where $T_{\omega }$ is the characteristic temperature and *p* is a free-fitting parameter. It should be noted that for some *R*(*T*) curves analyzed below we used a fixed *p* = 5 value in Equation (2), and for these cases the designation of $T_{\theta }$ is kept for the Debye temperature designation.

A primary idea is to utilize Equation (2) to reveal the type of charge carrier interaction, based on the well-established theoretical result that *p* in Equation (2) approaches unique integer values for different interaction mechanisms [47,48,49,50] (see Table 1).

This means that the normal part of the *R*(*T*) curve has a different shape for each charge carrier interaction mechanism, and this mechanism can potentially be revealed by the fit of *R*(*T*) data to Equation (2). However, because there is no expectation that real world material can exhibit only one interaction mechanism, the deduced *p*-value is the integrated value for all interaction mechanisms. Based on this, there is no expectation that the electron–phonon mechanism (manifested by *p* = 5) does not exist in materials with very strong electron–electron interaction (which is manifested by *p* = 2). As a result, the deduced *p*-value will be above *p* = 2, because of the partial contribution of the electron–phonon interaction with *p* = 5 (or partial contribution of the electron–magnon interaction with *p* = 3).

However, pure electron–phonon cases for elemental copper and silver, and also high-entropy alloy (ScZrNb)_0.65_[RhPd]_0.35_ (i.e., free-fitting p≅5), for which deduced $T_{\theta }$ is very close to the values measured by independent techniques, and pure electron–magnon cases for elemental iron and Sr_2_Cr_3_As_2_O_2_ (i.e., free-fitting p≅3) can be found in Refs. [44,45,46]. 

Thus, the dominant charge carrier interaction mechanism in the given materials can be determined from the comparison of the deduced free-fitting parameter *p* with the theoretical values for pure cases (Table 1). Because all considered *R*(*T*) datasets were measured for superconductors, we used the recently proposed equation in [44,46] to fit the full *R*(*T*) curve, including the superconducting transition:

(3) $$\begin{array}{cc} R\left(T\right)=R_{0}+\theta \left(T_{c}^{onset}-T\right) & \cdot \left(\frac{R_{norm}}{{\left(I_{0}\left(F\cdot {\left(1-\frac{T}{T_{c}^{onset}}\right)}^{3/2}\right)\right)}^{2}}\right)+\theta \left(T-T_{c}^{onset}\right)\\ & \cdot (R_{norm}+A\\ & \cdot \left({\left(\frac{T}{T_{\omega }}\right)}^{p}\cdot \overset{\frac{T_{\omega }}{T}}{\underset{0}{\int }}\frac{x^{p}}{\left(e^{x}-1\right)\cdot \left(1-e^{-x}\right)}\cdot dx-{\left(\frac{T_{c}^{onset}}{T_{\omega }}\right)}^{p}\cdot \overset{\frac{T_{\omega }}{T_{c}^{onset}}}{\underset{0}{\int }}\frac{x^{p}}{\left(e^{x}-1\right)\cdot \left(1-e^{-x}\right)}\cdot dx\right)) \end{array}$$

where $T_{c}^{onset}$ is the free-fitting parameter of the onset of superconducting transition, *R*_norm_ is the sample resistance at the onset of the transition, $\theta \left(x\right)$ is the Heaviside step function, *I*_0_(x) is the zero-order modified Bessel function of the first kind, and *F* is a free-fitting dimensionless parameter. *R*(*T*) data-fits to Equations (2) and (3) have been performed by using the Levenberg–Marquardt algorithm in the non-linear fitting package of the Origin software (version Origin2017, OriginLab Corp., Northampton, MA, USA) package.

## 3. Results

### 3.1. Highly Compressed Lithium

**Figure 1:**
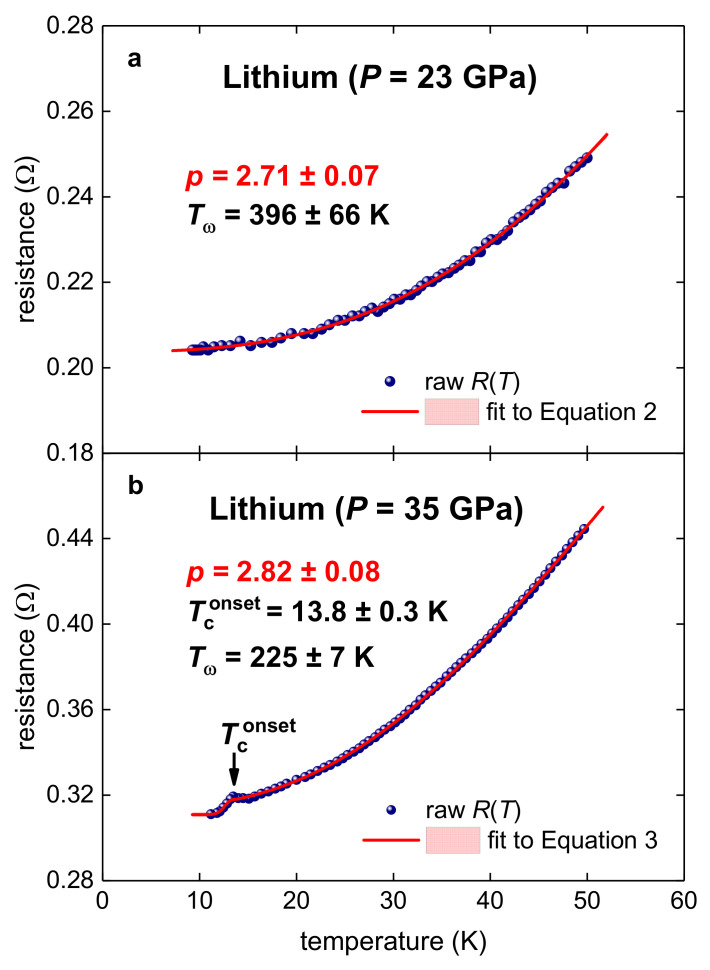
Resistance data, *R*(*T*), and fits to (**a**) Equation (2) and (**b**) Equation (3) for highly compressed lithium (raw *R*(*T*) data reported by Shimizu et al. [20]). (**a**)—deduced p=2.71±0.07 and $T_{\omega }=396\pm 66\;K$; the fit quality is 0.9990. (**b**)—deduced p=2.82±0.08, $T_{c}^{onset}=13.8\pm 0.3\;K$, $T_{\omega }=225\pm 7\;K$; the fit quality is 0.9998. Confidence bands at 95% are shown by pink shaded areas.

Shimizu et al. [20] (in their Figure 2) reported *R*(*T*) curves for lithium compressed at *P* = 3.5, 23, 35 and 36 GPa. Due to the overlapping of *R*(*T*) curves at *P* = 35 GPa and *P* = 36 GPa, we fitted *R*(*T*) datasets measured at *P* = 23 and 35 GPa, as shown in Figure 1. It can be seen in Figure 1 that the deduced *p*-value for both pressures are remarkably close to each other (i.e., p=2.7–2.8).

The main result of the analysis, i.e., p=2.7–2.8, implies that the many-fold disagreement between observed *T*_c_ and calculated *T*_c_ (as derived from first-principles calculations [18,19,26]) has the natural explanation that the charge carrier pairing in highly compressed lithium does not belong to the electron–phonon interaction.

### 3.2. Highly Compressed Black Phosphorous

**Figure 2:**
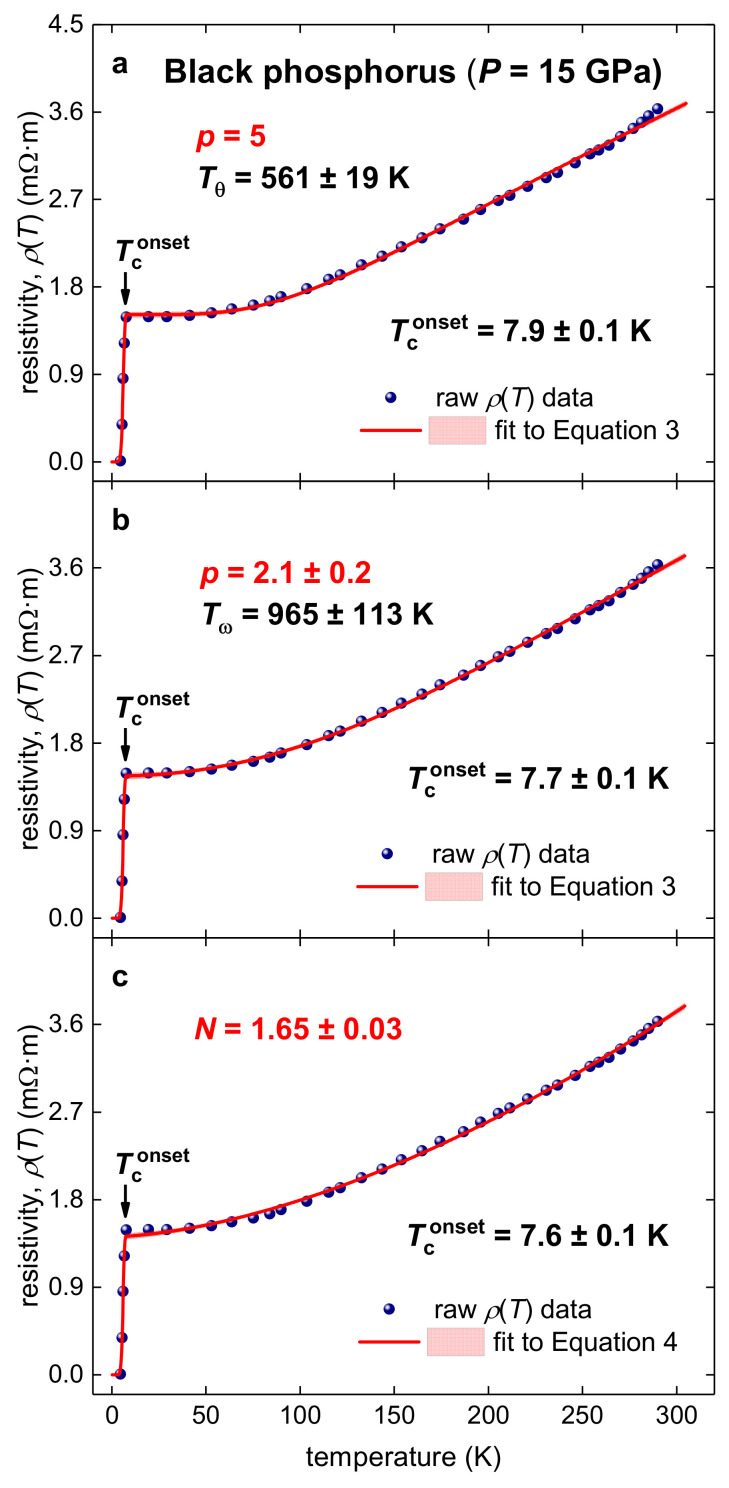
Resistivity data, *ρ*(*T*), and data-fits for Equation (3) for highly compressed black phosphorus (raw data is from Reference [51]). (**a**)—fit to Equation (3), *p* = 5, deduced $T_{\theta }=561\pm 19\;K$, $T_{c}^{onset}=7.9\pm 0.1\;K$, fit quality is 0.9986; (**b**)—fit to Equation (3), deduced p=2.1±0.2, $T_{\omega }=965\pm 113\;K$, $T_{c}^{onset}=7.7\pm 0.1\;K$, fit quality is 0.9992; (**c**)—fit to Equation (4), deduced N=1.65±0.03, $T_{c}^{onset}=7.6\pm 0.1\;K$, fit quality is 0.9989. Confidence bands at 95% are shown by pink shadow areas.

**Figure 3:**
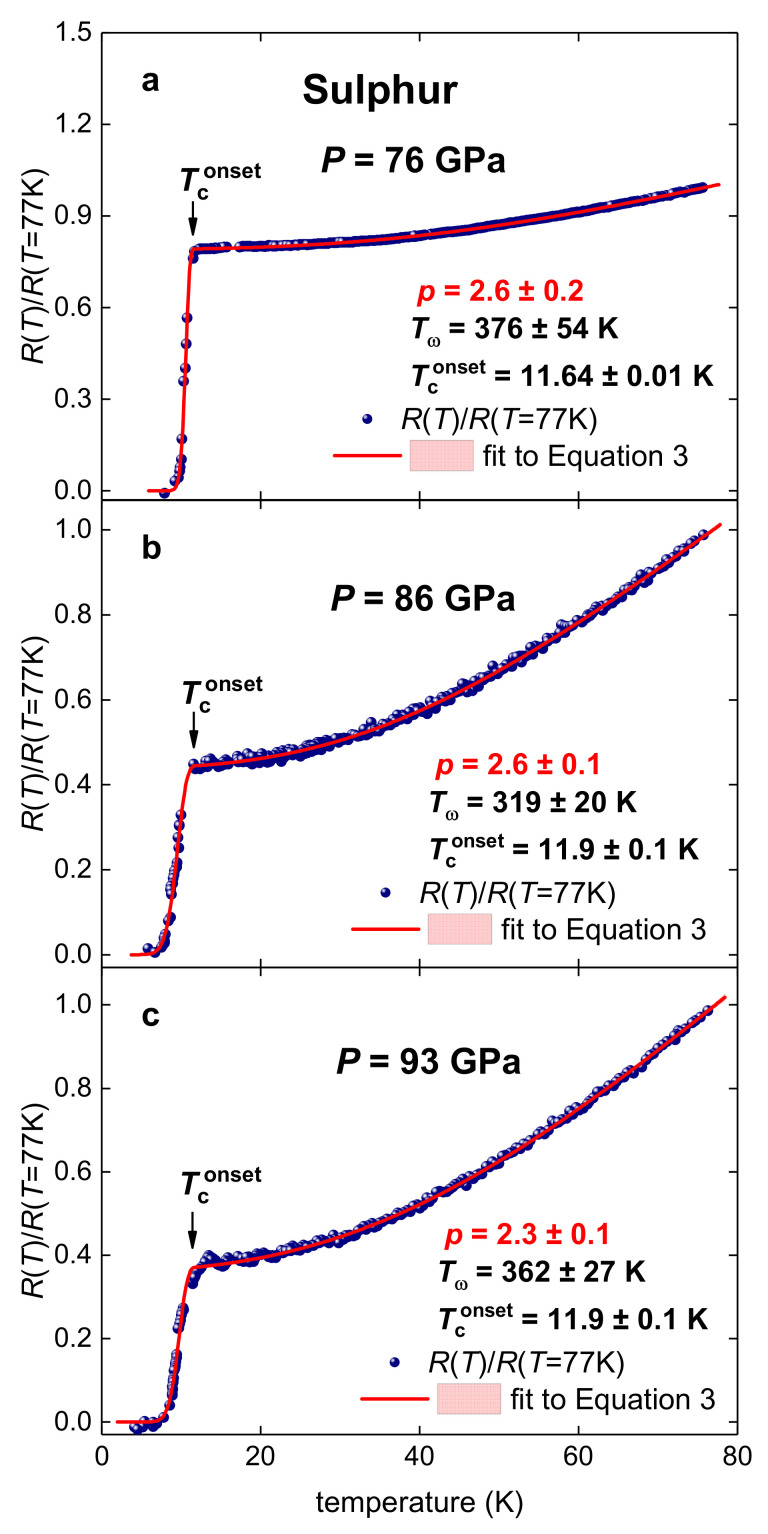
*R*(*T*)/*R*(*T* = 77 K) datasets for highly compressed sulfur (raw data is from Reference [22]) and fits to Equation (3) at *p* being a free-fitting parameter. (**a**)—*P* = 76 GPa, deduced p=2.6±0.2, $T_{\omega }=376\pm 54\;K$, $T_{c}^{onset}=11.64\pm 0.01\;K$, fit quality is 0.9979; (**b**)—*P* = 86 GPa, deduced p=2.6±0.1, $T_{\omega }=319\pm 20\;K$, $T_{c}^{onset}=11.9\pm 0.1\;K$, fit quality is 0.9982, (**c**)—*P* = 93 GPa, deduced p=2.3±0.1, $T_{\omega }=362\pm 27\;K$, $T_{c}^{onset}=11.9\pm 0.1\;K$, fit quality is 0.9985. Confidence bands at 95% are shown by pink shadow areas.

Shirotani et al. [51] in their Figure 5 reported the *ρ*(*T*) curve for black phosphorous compressed at *P* = 15 GPa. In Figure 2 (panels *a* and *b*), we fitted this dataset to Equation (3), where, in panel (a), *p* was fixed to 5, and in panel (b), *p* was a free-fitting parameter. It should be stressed that the goal of the analysis is not to obtain a fitting curve which approximates the experimental *R*(*T*) dataset with the highest possible quality. This is because, as we show in Figure 3c, a power-law fitting function:

(4) $$R\left(T,B\right)=R_{0}+\theta \left(T_{c}^{onset}-T\right)\cdot \left(\frac{R_{norm}}{{\left(I_{0}\left(F\cdot {\left(1-\frac{T}{T_{c}^{onset}}\right)}^{3/2}\right)\right)}^{2}}\right)+\theta \left(T-T_{c}^{onset}\right)\;\cdot \left(R_{norm}+A_{N}\cdot \left(T^{N}-{\left(T_{c}^{onset}\right)}^{N}\right)\right)$$

where *N* is free-fitting parameter, can fit the data with even higher quality than Equation (3) at the *p* = 5 (fixed) value (Figure 3a).

Instead, the goal of the fit to reveal the nature of the charge carrier interaction. For instance, deduced $T_{\theta }=561\pm 19\;K$ (Figure 2a) has a very clear physical interpretation. At the same time, the deduced value N=1.65±0.03 (Figure 2c) has no any meaningful interpretation, as recently showed in Reference [46]. Moreover, since our primary purpose is to reveal the dominant charge carrier interaction mechanism, it cannot be revealed when *p* = 5 (fixed), because this condition means that the data analysis is performed with assumption of pure electron–phonon charge carrier interaction. Thus, one way to reveal the charge carrier interaction is to assume that *p* is a free fitting parameter, and to deduce the value for this parameter (see, for instance, Figure 1 and Figure 2b).

At the condition of *p* = 5 (fixed), the deduced $T_{\theta }=561\pm 19\;K$ value implies the weak-coupling scenario in this superconductor within the electron–phonon pairing mechanism (because $\frac{T_{c}^{onset}}{T_{\theta }}≅0.01$). When *p* is a free-fitting parameter, its deduced value, p=2.1±0.2, unavoidably indicates the dominance of the electron–electron interaction in this highly compressed superconductor.

### 3.3. Highly Compressed Sulphur

Yakovlev et al. [28] reported on the observation of superconductivity in highly compressed sulphur, which became the first non-conductive element converted into a superconductor by applying high pressure. Here, in Figure 3, we fitted temperature dependent resistance data, $\frac{R\left(T\right)}{R\left(T=77\;K\right)}$, measured at *P* = 76, 86, and 93 GPa by Shimizu et al. [22] (raw data is shown in Figure 10 of Reference [22]).

These datasets were recently fitted to Equation (3) at fixed *p* = 5 by Talantsev and Stolze [50] and, thus, the fit quality and deduced $T_{\theta }$ at *p* = 5 can be found in Reference [39]. Free-fitting parameters *p* and $T_{\omega }$, deduced from the fits (Figure 3), are vary in the narrow ranges of p=2.5–2.8 and $T_{\omega }=319–376\;K$. Deduced *p* strongly implies that the charge carrier in highly compressed sulphur exhibits non-electron–phonon interaction. 

### 3.4. Highly Compressed Silane

**Figure 4:**
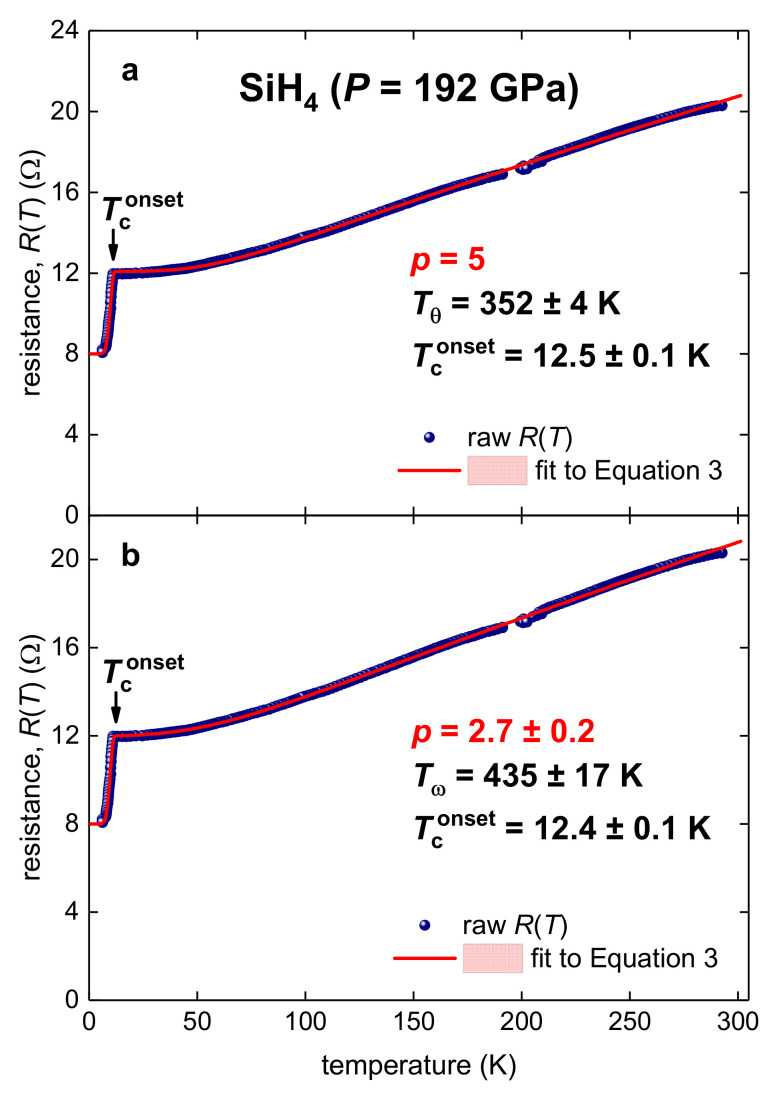
*R*(*T*) data and fits to Equation 3 for highly compressed silane (*P* = 192 GPa) (experimental data digitized from Figure 2b in Reference [12]). (**a**)—*p* = 5, $T_{\theta }=352\pm 4\;K$, $T_{c}^{onset}=12.5\pm 0.1\;K$, fit quality is 0.9995; (**b**)—deduced p=2.7±0.2, $T_{\omega }=435\pm 17\;K$, $T_{c}^{onset}=12.4\pm 0.1\;K$, fit quality is 0.9996. The fitting curves are red; confidence bands at 95% are shown by a pink shaded area.

As discussed above, highly compressed silane, SiH_4_, is one of the most challenging cases to the widely accepted paradigm that superconductivity in highly compressed hydrogen-rich compounds originates from the electron–phonon pairing mechanism. In Figure 4, we show *R*(*T*) data as reported by Eremets et al. [12] for SiH_4_ compressed at *P* = 192 GPa (in their Figure 2b), as well as data-fits to Equation (3) at *p* = 5 (panel a) and *p* as a free-fitting parameter (panel b). Despite the fact that, at *p* = 5, the fit converged and has high quality, when *p* is free-fitting parameter its value is p=2.7±0.2, and the deduced free-fitting characteristic temperature is $T_{\omega }=435\pm 17\;K$, and $T_{c}^{onset}=12.4\pm 0.1\;K$.

The first outcome of our analysis is that neither the deduced $T_{\theta }=352\pm 4\;K$, nor $T_{\omega }=435\pm 17\;K$, are close to the value, calculated by Feng et al. [11], of the Debye temperature of $T_{\theta }=3500-4000\;K$. The second outcome is that the free-fitting parameter value of p=2.7±0.2 implies the non-electron–phonon charge carrier interaction in this highly compressed hydrogen-rich compound.

## 4. Discussion

Due to the fact that the analysis via Equations (2) and (3) has only developed very recently [43,44,45], there is a need to discuss the limitations of the analysis. It should be stressed that the key element of the analysis is the integral term in the Equation (3):

(5) $${\left(\frac{T}{T_{\omega }}\right)}^{p}\cdot \overset{\frac{T_{\omega }}{T}}{\underset{0}{\int }}\frac{x^{p}}{\left(e^{x}-1\right)\cdot \left(1-e^{-x}\right)}\cdot dx$$

and, thus, to be properly analyzed by Equation (3), the raw experimental *R*(*T*) dataset should be measured at a reasonably wide temperature range:

(6) $$\frac{T_{c}}{T_{\omega }}<\frac{T}{T_{\omega }}<\frac{T_{upper\;bond}}{T_{\omega }}$$

where, in an ideal case:

(7) $$T_{upper\;bond}\geq T_{\omega }$$

However, because overall (with only a few exceptions; see, for instance Reference [52]) there was no intention to use the normal part of *R*(*T*) datasets to deduce the Debye temperature of highly compressed superconductors (including NRTS materials [50]), measurements of *R*(*T*) datasets were performed in ranges approximately 20–40 K above the transition temperature. Thus, there is only a very limited number of reported *R*(*T*) datasets for highly compressed superconductors which can be fitted to Equation (3), when *p* is a free-fitting parameter.

Even if *p* is fixed to *p* = 5 (which implies that deduced values belong to the electron–phonon interaction), the *R*(*T*) datasets should be measured at a reasonably wide temperature range, because the fit is based on the calculation of the integral (Equation (5)), and accurate calculation of the integral requires thousands or, in some exceptional cases, up to 35,000 raw *R*(*T*) datapoints [5,16]. 

## 5. Conclusions

In this paper, we analyzed *R*(*T*) data for the highly compressed elemental superconductors lithium, black phosphorous, and sulfur, and also for simple hydrogen-rich silane, SiH_4_. Overall, all studied superconductors exhibit very close values for parameter *p* in the generalized Bloch–Grüneisen (BG) equation (Equations (2) and (3)), which vary within a narrow range of p=2.0–2.8. This range of *p* is very different from p=5 (i.e., the unique characteristic value belonging to the electron–phonon charge carrier interaction). This result is in good accord with our earlier result [44], where we reported essentially the same deduced values of p=1.8–3.2 for highly compressed boron, H_3_S, LaH_x_, PrH_9_ and BaH_12_. 

Overall this implies that non-electron–phonon mechanisms, and particularly the electron–electron retraction, should be considered as an alternative possibility to be the origin for the emergence of the superconductivity in highly compressed materials.

## Figures and Tables

**Table 1:** *p*-value in generalized Bloch–Grüneisen equation (Equation (2)) and the interation mechanism designated for that value [47,48,49,50].

*p*	Charge Carrier Interaction Mechanism	Integral Term in Equation (2)
2	the electron–electron interaction	${\left(\frac{T}{T_{\omega }}\right)}^{2}\cdot \overset{\frac{T_{\omega }}{T}}{\underset{0}{\int }}\frac{x^{2}}{\left(e^{x}-1\right)\cdot \left(1-e^{-x}\right)}\cdot dx$
3	the electron–magnon interaction	${\left(\frac{T}{T_{\omega }}\right)}^{3}\cdot \overset{\frac{T_{\omega }}{T}}{\underset{0}{\int }}\frac{x^{3}}{\left(e^{x}-1\right)\cdot \left(1-e^{-x}\right)}\cdot dx$
5	the electron–phonon interaction	${\left(\frac{T}{T_{\omega }}\right)}^{5}\cdot \overset{\frac{T_{\omega }}{T}}{\underset{0}{\int }}\frac{x^{5}}{\left(e^{x}-1\right)\cdot \left(1-e^{-x}\right)}\cdot dx$

## Data Availability

No new data were created or analyzed in this study. Data sharing is not applicable to this article.
